# Smyd1 Facilitates Heart Development by Antagonizing Oxidative and ER Stress Responses

**DOI:** 10.1371/journal.pone.0121765

**Published:** 2015-03-24

**Authors:** Tara L. Rasmussen, Yanlin Ma, Chong Yon Park, June Harriss, Stephanie A. Pierce, Joseph D. Dekker, Nicolas Valenzuela, Deepak Srivastava, Robert J. Schwartz, M. David Stewart, Haley O. Tucker

**Affiliations:** 1 Molecular Biosciences and Institute for Cellular and Molecular Biology, University of Texas, Austin, Texas, United States of America; 2 Hainan Provincial Key Laboratory for Human Reproductive Medicine and Genetic Research, Affiliated Hospital of Hainan Medical University, Haikou, Hainan, P.R. China; 3 Department of Biology and Biochemistry, University of Houston, Houston, Texas, United States of America; 4 Gladstone Institute of Cardiovascular Disease and Departments of Pediatrics and Biochemistry and Biophysics, University of California, San Francisco, California, United States of America; University of Houston, UNITED STATES

## Abstract

Smyd1/Bop is an evolutionary conserved histone methyltransferase previously shown by conventional knockout to be critical for embryonic heart development. To further explore the mechanism(s) in a cell autonomous context, we conditionally ablated *Smyd1* in the first and second heart fields of mice using a knock-in (KI) *Nkx2*.*5-cre* driver. Robust deletion of *floxed-Smyd1* in cardiomyocytes and the outflow tract (OFT) resulted in embryonic lethality at E9.5, truncation of the OFT and right ventricle, and additional defects consistent with impaired expansion and proliferation of the second heart field (SHF). Using a transgenic (Tg) *Nkx2*.*5-cre* driver previously shown to not delete in the SHF and OFT, early embryonic lethality was bypassed and both ventricular chambers were formed; however, reduced cardiomyocyte proliferation and other heart defects resulted in later embryonic death at E11.5-12.5. Proliferative impairment prior to both early and mid-gestational lethality was accompanied by dysregulation of transcripts critical for endoplasmic reticulum (ER) stress. Mid-gestational death was also associated with impairment of oxidative stress defense—a phenotype highly similar to the previously characterized knockout of the Smyd1-interacting transcription factor, skNAC. We describe a potential feedback mechanism in which the stress response factor Tribbles3/TRB3, when directly methylated by Smyd1, acts as a co-repressor of Smyd1-mediated transcription. Our findings suggest that Smyd1 is required for maintaining cardiomyocyte proliferation at minimally two different embryonic heart developmental stages, and its loss leads to linked stress responses that signal ensuing lethality.

## Introduction

Cardiac organogenesis initiates in the cardiac crescent at embryonic day (E) 7.75 and gives rise to the linear heart tube by E8.0 [1]. Cardiac progenitor cells, termed the anterior/second heart field (SHF), originate in splanchnic mesoderm and contribute to the linear heart tube as it undergoes morphogenesis into a full four chambered heart [1]. A spectrum of congenital heart anomalies result in humans from perturbation of SHF formation, including atrial and conotruncal defects [2]. An expanding network of transcription factors, such as ISL LIM Homeobox 1 (Isl1), Hand 2 [3], and Myocyte Enhancer Factor 2C (Mef2c), as well as intercellular signaling pathways control SHF deployment by controlling cardiac progenitor cell proliferation in pharyngeal mesoderm, progenitor cell differentiation, and cardiac progenitor cell patterning in the dorsal pericardial wall [1]. Coordination of these three processes in the early embryo drives progressive heart tube elongation during cardiac morphogenesis.

The subsequent maturation of the heart and outflow tract (OFT) is a tightly regulated process in which oxygen tension plays a vital role [4]. It is now well established that the developing embryo is very sensitive to oxidative stress by the production of reactive oxygen species (ROS) which may induce embryonic death and malformations, such as fetal intrauterine growth retardation [5]. The accumulation of misfolded or unfolded proteins in response to ROS-mediated oxidative stress can trigger endoplasmic reticulum (ER) stress as an adaptive cellular response. Cation transport regulator homolog 1 (Chac1) and cEBP homologous protein (Chop/GADD153) are considered to be vital initiators of the ER stress response [6,7]. Oxidative stress as well as a variety of other signals that induce ER stress can stimulate either apoptosis or autophagy in order to clear damaged cells [6]. It is unclear what regulates the balance between these two clearance processes. At midgestation, anti-oxidant defense is initiated by expression of Myoglobin (*Mb*) and associated factors that contribute to oxygen storage and delivery [8]. Skeletal NAC (skNAC), a skeletal and heart pre-mRNA splice variant of *nascent polypeptide-associated complex alpha subunit (αNAC)*, was shown to act as a muscle-specific transcriptional activator of *Mb* [9], and ∼50% of *skNAC*-deficient mice die between E9.5 and E12.5 due to cardiac malformations. At the molecular level, these mutant embryos have decreased expression of *Mb* and other anti-oxidative stress factors.

*SET-MYND-domain 1 (Smyd1/Bop)* encodes an evolutionary conserved histone methyltransferase containing a split SET (Su(var), Enhancer-of-zeste, Trithorax) domain interrupted by a MYND (Myeloid, Nervy and DEAF-1) domain [10] and physically interacts with skNAC [11,12]. This interaction occurs through the zinc finger MYND domain, a well characterized protein-protein interaction motif known best for its association with co-repressor complexes [13,14,15]. Expression of the *Smyd1* gene is restricted to muscle tissues in human, fish, frog, chicken and mouse [10,16,17,18]. Global knockdown of *Smyd1a and Smyd1b* in zebrafish resulted in the disruption of myofibril formation and an absence of beating in the heart [16]. The zebrafish mutant *flatline (fla)*, which harbors a nonsense mutation in the *Smyd1b* gene, exhibits a major defect in thick filament assembly causing both cardiac and skeletal muscle dysfunction [19]. Conventional null *Smyd1* mice die *in utero* at E9.5 due to heart defects, including disrupted maturation of ventricular cardiomyocytes and malformation of the right ventricle (RV) [10]. Supporting a role for Smyd1 in muscle development, Smyd1 induced myocyte differentiation when expressed in C2C12 myoblasts [18].

Smyd1 has been shown to function through multiple mechanisms, any or all of which could be causal for the above described phenotypes. Firstly, Smyd1 functions as repressor through interaction of its MYND domain with co-repressors HDAC1–3, NCoR and SMRT [11]. Secondly, Smyd1 functions as a transcriptional activator by catalyzing trimethylation of H3K4 [16], a histone modification associated with transcriptionally active loci [20]. Lastly, Smyd1 interacts and colocalizes with skNAC in skeletal and heart muscle [11,12], but the relevance of the interaction is unknown. While the highly related paralogue Smyd2 methylates a number of non-histone targets [21,22,23], such has not been described for Smyd1.

During mouse embryonic development, expression of *Smyd1* is detected as early as E7.75 in the cardiac crescent and the SHF, and later expression is found in the developing heart tube and OFT [10]. Expression continues throughout development and into adulthood. Thus, we reasoned that Smyd1 regulates gene program(s) central to both early and late heart development. To test this hypothesis, we generated mice harboring a conditional knockout (CKO) allele for *Smyd1 (Smyd1* ^*flox*^) and crossed them to two different cardiomyocyte-specific *Nkx2*.*5-cre* mouse lines: (1) a knock-in, in which the *cre* open reading frame was inserted into the first exon of the *Nkx2*.*5* gene (*Ki-Nkx2*.*5* ^*cre/+*^) previously shown to delete in the SHF [24] and (2) a transgenic, in which *cre* is expressed under the control of the *Nkx2*.*5* basal promoter and cardiac enhancer (*Tg-Nkx2*.*5-cre*) shown to delete subsequent to SHF and OFT development [25]. Early deletion using *Ki-Nkx2*.*5* ^*cre/+*^ resulted in E9.5 death, with defects consistent with impaired expansion of the SHF and OFT. Deletion with *Tg-Nkx2*.*5-cre* led to delayed lethality (E11.5–12.5) and to defects akin to those observed in *skNAC* knockout mice [12]. Proliferative defects underlying both phenotypes led to induction of ER and oxidative stress programs. Among the up-regulated genes, a sensor of both stress responses and a Smyd1-interacting protein, Tribbles3/TRB3 [26,27], was directly methylated by Smyd1. Methylated TRB3, in turn, functions as a Smyd1 co-repressor, suggestive of a potential feedback loop for stress response transcription.

## Materials and Methods

### Animals

*Smyd1 flox* mice were generated using endogenous sequence obtained from a Lambda FixII Vector 129SV Mouse Genomic Library (Stratagene). The targeting construct was designed in the Osdupdel vector, a gift from William A Kuziel, in order to introduce *loxP* sites flanking the second and the third exons of *Smyd1* followed by a neomycin cassette that is flanked by a third *loxP* site. The targeting vector was electroporated into 129S6 ES cells. After selecting with G418 and gancyclovir, surviving clones were screened for homologous recombination by Southern blot after digestion either with Bgl II and Sal I or Bgl II and Kpn I, and hybridization with probe 2 and probe 3, respectively. ES clones showing correct targeting in both arms were injected into C57BL/6 blastocysts to create chimeric mice. The chimeric mice were mated to C57BL/6 females to create a germline knock-in referred to as *Smyd1* ^*FloxNeo*^. *Smyd1* ^*FloxNeo*^ mice were crossed to *EIIa-cre* mice, in which cre is expressed at the 2-cell stage of development (The Jackson Laboratory, #003724) and progeny were screened for deletion of the neomycin cassette by Southern blot and back-crossed in order to remove *cre* resulting in germline *Smyd1* ^Flox^ mice. From these crosses, some mice were also generated that had ubiquitous and germline deletions of the *Smyd1* ^*FloxNeo*^ locus. These animals were termed *Smyd1* ^*Floxdel*^ and used to confirm that the germline deletion of *Smyd1* phenocopies the original conventional mutation (*Smyd1* ^*KO*^). Knock-in (*Ki)-Nkx2*.*5* cre and transgenic (*Tg)-Nkx2*.*5-cre* mice were previously described [24,25]. This study was carried out in strict accordance with the recommendations in the Guide for the Care and Use of Laboratory Animals of the National Institutes of Health. All experimental procedures involving mice were approved by the Institutional Animal Care and Use Committees of the University of Houston or the University of Texas at Austin.

### Whole-mount X-gal staining

Whole-mount staining for β-galactosidase (*lacZ*) activity was performed according to a standard protocol [28].

### Whole-mount RNA *in situ* hybridization

Whole-mount RNA *in situ* hybridization was carried out as previously described [29]. *Isl1* and *Mef2c* probes were kindly provided by Dr. James Martin (Baylor College of Medicine, Houston, TX). Complementary RNA probes for *Anf*, *Hand2*, *Myh7 (β-MHC)* and *Smyd1* were generated previously [30]. Detailed probe information is available upon request.

### Real-time PCR and microarrays

Hearts and pharyngeal arches were collected from E9.5 control and *Smyd1* Ki-CKO embryos. 20 hearts were pooled for each experiment. Individual hearts were collected and assayed from E10.5 control and Smyd1 Tg-CKO embryos. RNA was isolated with a Micro RNeasy kit (Qiagen, Venlo, Netherlands), and treated with RNase-free DNase I (Roche, Basel, Switzerland) to remove genomic DNA. RNA (1 μg or more) was sent to Phalanx Biotech (Palo Alto, CA) for Ambion dual amplification followed by genome-wide gene expression analysis. The raw data were normalized and analyzed using OneArray Studio software (Phalanx Biotech). Ratios were determined from three independent experiments and each sample was hybridized to duplicate microarrays. Complementary DNA was prepared from 1 ug total RNA using AMV Reverse Transcriptase (Invitrogen, Carlsbad, CA) following the manufacturer’s protocol. Real-time PCR analysis for *Isl1*, *Gapdh* and *Mef2c* was performed using Taqman probes. Real-time PCR analysis for *Hand2*, *Myh7 (β-Mhc)*, *Gapdh* and *Anf* was carried out using Brilliant qPCR reagent and the Mx3000P Real-Time PCR System (Stratagene, La Jolla, CA). Primers were generated in our lab and sequences are available upon request. Ratios were determined from three independent experiments, each evaluated in triplicate by real-time PCR.

### Histological analysis

Fixed tissues were cryopreserved and embedded in OCT before staining with H&E using standard techniques or anti-BrdU (DSHB clone G3G4; 1:250) and anti-mouse–DAB secondary sera. Paraffin embedded sections were stained with H&E using standard protocols or with anti-phospho-histone 3 S10 (H3S10ph) (Millipore, 1:100) and anti-rabbit-FITC secondary sera.

### Expression plasmids

Mammalian expression plasmids were constructed in the backbone vector PCDNA3.1 containing either a MYC or FLAG tag. Construction of the following TRB3 protein truncations were constructed by PCR using EcoRI and XhoI restriction sites: TRB3 (1–80); TRB3 (1–149); TRB3 (1–214); TRB3 (1–295); TRB3 (81–355); TRB3 (150–355); TRB3 (215–355); TRB3(296–355). All PCR amplifications were performed with high fidelity Pfu Turbo DNA polymerase (Stratagene) and sequenced for accuracy. Construction of the catalytic mutant Smyd1-Y234 and TRB3 mutants K16/R, K17R, K33R and P45/G were constructed by QuikChange Site-Directed Mutagenesis (Stratagene). GAL4-Smyd1 and SV40 promoter-driven 5X-GAL4DBD-luciferase were previously reported [10]. For bacterial expression (6X-His)-tagged-full-length Smyd1, TRB3 and their respective catalytic, deletion and point mutations were cloned into Gateway (Invitrogen) pET-DEST42. High-level expression was induced by IPTG in E. coli strains MG232 (Scarab LTM) or Hsp90PlusTM (Expression Technologies Inc.). Proteins were purified to near homogeneity by Ni-bead chromatography as detailed in the Scarab LTM protocol.

### Immunoprecipitation and western blotting

Cell lysates were incubated with 10 ml of 50% slurry of protein-A/G sepharose beads for 1 h to preclear. One microgram of polyclonal MYC A14 or monoclonal FLAG M2 (Sigma) antibody was added to the supernatant for a minimum of 4 hours at 4°C, followed by a 1 hour incubation with 20 ml protein-A/G sepharose beads (Sigma). After four washes with lysis buffer, the precipitated proteins were analyzed by SDS-PAGE and transferred to PVDF membrane. Membranes were probed with the monoclonal FLAG M2 or monoclonal MYC9E10. Western blots were developed with the enhanced chemiluminescence (ECL) analysis system (GE Life Sciences).

### Cell culture and transient transfections

Cos7 cells (ATCC) were grown in DMEM (Life Technologies, Frederick, MD) supplemented with 10% fetal bovine serum at 37° C in an atmosphere of 5% CO_2_ in air. The cells were transfected using FuGENE6 reagent (Roche) or Lipofectamine Plus (Invitrogen). For luciferase assays, the total DNA concentration was held constant by adding the empty PCDNA3.1 expression plasmid. Cells were harvested 30–36 hours after transfection using the lysis buffer contained in the Promega Luciferase Kit. For immunoprecipitation experiments, Cos7 cells were transfected with FLAG-tagged Smyd1 or with FLAG-tagged Smyd1 and MYC-tagged TRB3 deletion constructs. Cells were lysed in buffer (10 mM Tris pH 7.4, 1mM EDTA, pH 8.0, 0.5% Triton X100 in PBS) containing protease inhibitors (Roche) and PMSF 30–36 hours after transfection.

### Luciferase assays

Cell lysates were analyzed with a Rosys Anthos Lucy2 luminometer. Relative light units obtained from the pGL2-5XGAL4-SV40 reporter (Luciferase Assay Kit, Promega) were normalized by spectrophotometric analysis of cells co-transfected with the RSV-LacZ expression plasmid. Activation was determined in relation to the pGL2-5XGAL4-SV40 reporter alone. Three independent experiments were performed to calculate the mean and standard error.

### Lysine methyltransferase assays

The *in vitro* reactions were performed as previously described [31]. Briefly, ∼μ1 g bacterially purified 6XHis-Smyd1, 6XHis-Smyd1or 6XHis-Set2 (the kind gift of Dr. Danny Reinberg) were incubated with 1 μg of TRB3 wild type and mutants. Approximately 2 μCi S-adenosyl-L–[methyl-^3^H] methionine (SAM; GE Biosciences) was included as a methyl donor. All reactions were carried out in 40μl HMT reaction buffer (10mM dithiothreitol, 100mM NaCl, 4mM MgCl_2_, and 50mM Tris-HCl at pH 8.8) at 30°C for 3 hours. A 12% SDS-PAGE gel was used to resolve the samples and fluorography was used to visualize positive methylation. Substrate loading was visualized by Coomassie blue staining.

*In vivo* methylation of TRB3 was performed with a procedure modified from the method of Liu et al. [32]. 48 hr post transfection of Smyd1 or Smyd1-Y234/F, three plates (100-mm-diameter) of C2C12 cells were incubated with cycloheximide (100 mg/ml) and chloramphenicol (40 mg/ml) in standard media. L-[*methyl*-3H]methionine was added to a concentration of 10 mCi per ml. Cells were then incubated for an additional 3 hr, and lysed in RIPA buffer (50 mM Tris at pH 8.0, 150 mM NaCl, 0.5% deoxycholate, 0.1% SDS, 1% NP-40, 2 mM NaF,2 mM NaOV4, protease inhibitor cocktail tablets [Roche Diagnostics] with 2x concentration). Endogenous TRB3 was immunoprecipitated with anti-TRB3 polyclonal antibody (Sigma-Aldrich, T8076), and immunoprecipitated proteins were resolved on 8% SDS-PAGE. [^3^H]Methyl labeling was detected by fluorography in 22% PPO solution. Dried gels were exposed to Kodak MRX film for 1 week.

### Computational analyses

A Student’s *t* test was used for statistical comparisons when appropriate. A *p-*value of < 0.05 was considered statistically significant. Global microarray analyses employed Nimblegen chips and were analyzed using Java Treeview and DAVID [32].

## Results

### Expression of *Smyd1* during early heart development

**Fig 1:**
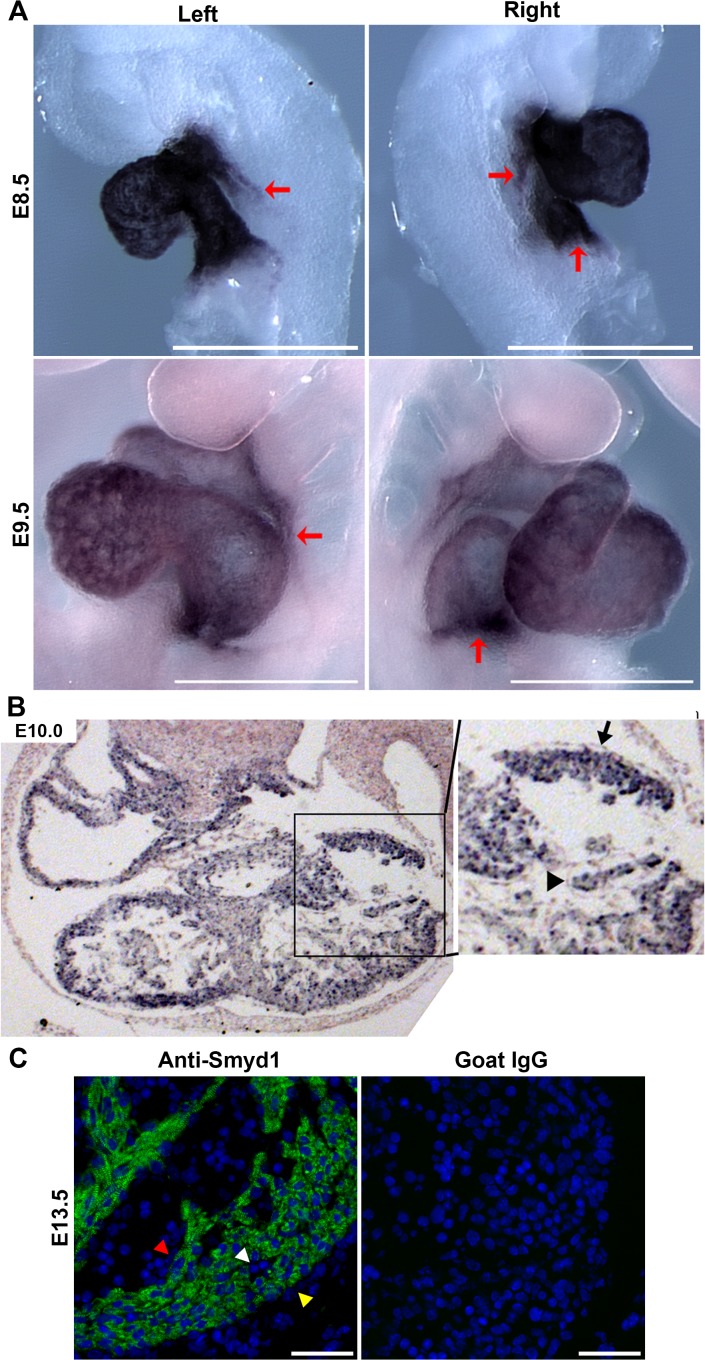
Expression of *Smyd1* during early heart development. **A**. Whole mount *in situ* hybridization for *Smyd1* in E8.5 (top panel) and E9.5 embryos (bottom panel) are shown from the left and right side. Red arrows denote the boundaries of *Smyd1* expression at the atrial and venous poles. Scale bar = 500 μm. **B.** *In situ* hybridization for *Smyd1* in transverse sections of E10.0 hearts. The box in the left panel is enlarged in the right panel. *Smyd1* mRNA was enriched in the myocardium and not detectable in either the epicardium or endocardium. Black arrow = epicardium; Black arrowhead = endocardium. **C.** Immunolocalization of Smyd1 protein in the heart at E13.5. Smyd1 was specifically detected in cardiomyocytes. No Smyd1 protein was detectable in the endocardium (red arrowhead), epicardium (yellow arrowhead) or coronary vasculature (white arrowhead). Scale bar = 50 μm.

To investigate the temporal and spatial expression of *Smyd1* during embryogenesis, we performed whole mount *in situ* hybridization on embryos recovered at E8.5 and E9.5. Results of this analysis demonstrated that at E8.5 and E9.5, robust expression of *Smyd1* was observed throughout the looped heart tube, including the ventricles, OFT and venous pole (Fig. 1A). To define which cell type(s) express *Smyd1*, we performed *in situ* hybridization on transverse sections at E10.0 and immunofluorescence on transverse sections at E13.5. *Smyd1* mRNA was enriched in the myocardium and undetectable in the endocardium or epicardium (Fig. 1B). Smyd1 protein was very specifically localized to cardiomyocytes and undetectable in the endocardium, epicardium or coronary vasculature. Within cardiomyocytes, Smyd1 protein was localized to the sarcoplasm, showing a sarcomeric pattern similar to that reported for zebrafish (Fig. 1C).

### Generation of *Smyd1* CKO mice

In order to analyze the role of Smyd1 specifically in the heart, we generated a *Smyd1* CKO allele using the targeting strategy schematized in S1A Fig. The *Smyd1* gene encodes three isoforms (*Smyd1a*, -*b*, and -*c*); *Smyd1c* utilizes an alternate first exon. Therefore, we designed this conditional allele with *loxP* sites flanking exons 2 and 3, the first 2 exons in common among all three isoforms and the same exons that were deleted in the conventional knockout [10]. Thus, in the presence of Cre recombinase, exons 2 and 3 are deleted resulting in a frameshift and a premature stop. Following validation of correct ES cell targeting and germline transmission (S1B-C Fig.), we confirmed that our deletion strategy was sufficient to replicate the conventional knockout by crossing *Smyd1* ^*flox/flox*^ with *EIIA-cre* [33], which is expressed in pre-implantation zygotes and leads to germline deletion (data not shown).

Next, we performed a cardiac-specific *Smyd1* CKO using the *Ki-Nkx2*.*5* ^*cre/+*^ strain previously shown to robustly delete in the first and SHF from E7.5 [24]. Examination of 21 litters from *Ki-Nkx2*.*5* ^*cre/+*^; *Smyd1* ^*+/flox*^ x *Smyd1* ^flox/flox^ revealed that 100% of *Ki-Nkx2*.*5* ^*cre/+*^; *Smyd1* ^*flox/flox*^ (henceforth referred to as *Smyd1 Ki-CKO*) embryos died prior to E10.5. In contrast, control littermates of all other genotypes survived with no apparent abnormalities. No transcripts from the deleted exons were detectable from *Smyd1 Ki-CKO* hearts at E9.5 (S1D Fig.). We also performed western blots for Smyd1 using an antibody raised against the C-terminus of Smyd1 and observed no protein product in heart extracts from Smyd1 CKO embryos at E9.5 (S1E Fig.).

### *Smyd1* Ki-CKO embryos exhibit malformation of SHF derivatives and alterations in SHF gene expression

**Fig 2:**
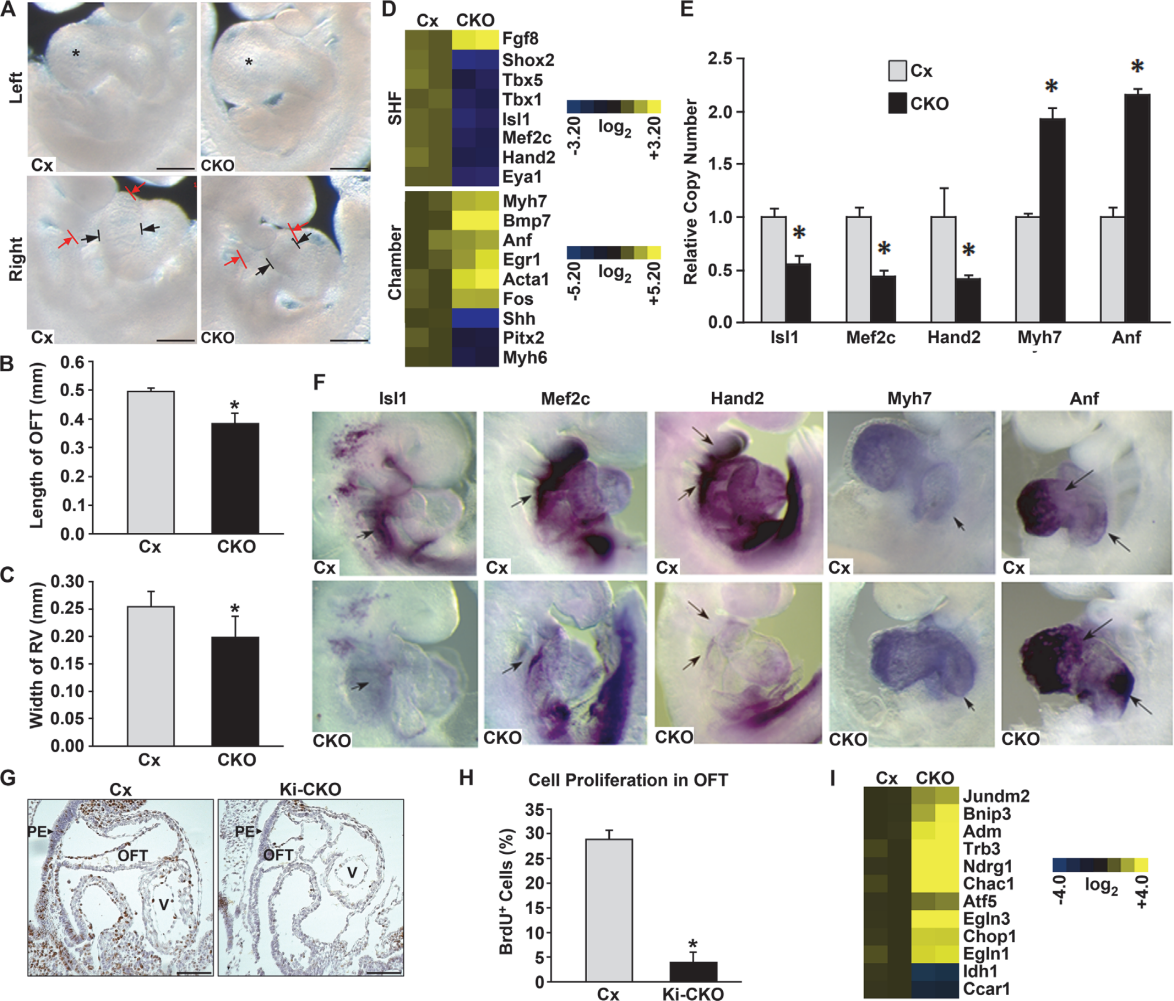
Loss of *Smyd1* using *Ki-Nkx2*.*5* ^*cre/+*^ disrupts looping morphogenesis and chamber formation through perturbation of the SHF and activation of ER stress. **A.** Gross morphological comparison of control (Cx: *Nkx2*.*5* ^*+/+*^; *Smyd1* ^*Flox/Flox*^) and *Smyd1* Ki-CKO (CKO: *Nkx2*.*5* ^*cre/+*^; *Smyd1* ^*Flox/Flox*^) hearts at E9.5. Scale bar = 200 μm. **B, C**. The lengths of the outflow tract (OFT) (B) and right ventricle (RV) (C) were significantly reduced in Ki-CKO embryos at E9.5 (n = 6/group). **D.** Representative results of microarray gene expression comparison of transcripts critical to SHF and chamber formation at E9.5. Data are presented as expression values of 2 independent biological replicas of each genotype averaged from 2 technical replicas. **E.** Confirmation of microarray for deregulated transcripts critical to SHF and chamber formation by real-time PCR using RNA from E9.5 heart/pharyngeal mesoderm (n = 9/group). **F**. Whole mount *in situ* hybridization comparison of selected SHF and chamber formation transcripts deregulated and/or mislocalized in CKO hearts at E9.5 (n = 3/group). Arrows denote areas of differential expression. **G.** Comparison of cell proliferation in the outflow tract of Control (Cx) and Ki-CKO by BrdU immunohistochemistry. PE, Pharyngeal Endoderm. OFT, outflow tract. V, ventricle. Scale bar = 100 μm. **H.** Quantification of anti-BrdU staining in the outflow tract (n = 6/group). **I.** Loss of Smyd1 leads to deregulation of genes critical to anti-proliferative responses to ER stress. Data are presented as a heat map with expression values of 2 independent biological replicas of each genotype averaged from 2 technical replicas plotted as log^2^ expression values. For B, C, E and H, data were analyzed by Student’s t-test (*P < 0.05).

At a gross morphological level, *Smyd1 Ki-CKO* embryos appeared to be slightly growth retarded at E9.5 (data not shown). The length of the OFT and width of the RV appeared truncated (Fig. 2A). Therefore, we measured the length of the OFT (Fig. 2B) and the width of the RV (Fig. 2C). Both were significantly smaller in the *Smyd1 Ki-CKO*.

Truncation of the OFT and RV indicated that *Smyd1* may be necessary for the expansion and maintenance of the SHF. To assess molecular mechanisms underlying these defects, we compared gene expression profiles of WT and *Smyd1* Ki-CKO hearts at E9.5 by microarray. Consistent with the phenotypic results, loss of *Smyd1* led to reduction in the expression of several factors required for proper development of SHF derivatives, including *Isl1*, *Mef2c*, *Hand2/dHAND*, *Tbx5*, *Tbx1*, *Shox2 and Eya1* (Fig. 2D, top panel). Reduction of *Hand2* expression is consistent with that of reported for the conventional *Smyd1* knockout [10]. *Fgf8* was modestly upregulated in *Smyd1* Ki-CKO hearts.

We also observed significant upregulation of genes involved in chamber formation and/or cardiac stress response, including *Myh7*, *Bmp7*, *Anf*, *Egr1*, *Acta1 and Fos*. In contrast, *Myh6* (*α-MHC*) and two genes crucial for establishing atrial-ventricle morphology and sidedness, *Shh* and *Pitx2* [34,35] were down-regulated in the *Smyd1* Ki-CKO (Fig. 2D, lower panel).

We confirmed these microarray results by real-time PCR using RNA extracted from E9.5 heart tissues. Expression of SHF transcription factor genes *Isl1*, *Mef2c* and *Hand2* was decreased. Expression of chamber-specific genes *Myh7* and *Anf* was increased (Fig. 2E).

Alterations in SHF and chamber gene expression were further assayed by whole mount *in situ* hybridization. Expression of *Isl1*, *Mef2c and Hand2* was reduced in *Smyd1* Ki-CKO hearts. Expression of *Anf* and *Myh7* was expanded into the AV canal and atrium (Fig. 2F). These data support the idea that Smyd1 plays a critical role in regulating the gene expression program governing early heart development.

### Loss of Smyd1 at E9.5 deregulates genes critical to anti-proliferative responses to ER stress

To determine whether the smaller OFT and RV in *Smyd1 Ki-CKO* embryos resulted from altered proliferation of SHF progenitors, we performed immunostaining for BrdU. At E9.5, *Smyd1* Ki-CKO embryos appeared to have less BrdU^+^ cells throughout the heart (Fig. 2G). As an indicator of SHF cell expansion, we quantified the percentage of BrdU^+^ cells in the OFT and found a significant decrease in cell proliferation for the Ki-CKO (Fig. 2H).

We further analyzed our microarray data for proliferation-related genes. These analyses indicated that cardiac ablation of *Smyd1* resulted in an increase in anti-proliferative genes, particularly those known to respond to ER stress by activating autophagic responses (Fig. 2I). These included BCL2/adenovirus E1B 19kDa interacting protein 3 (*Bnip3*), Activation Transcription Factor 5 (*Atf5*), and *Chop1*/GADD153. Simultaneously, genes canonically down-regulated during autophagy induced by ER-stress [36,37], such as Isocitrate Dehydrogenase 1 (*Idh1*) and Cell Cycle and Apoptosis Regulatory Protein 1 (*Ccar1*), were down-regulated. Furthermore, genes known to be unregulated in cardiomyocytes by both ER stress and hypoxia, such as Prolyl hydroxylase domain 1 and 3 *(PHD/Egln1/3)*, Jun dimerization protein 2 (*Jundm2/JDP2*), adrenomedullin (*Adm*), and Tribbles 3 (*TRB3*) [26,27,38,39,40,41], are overexpressed in the *Smyd1* Ki-CKO (Fig. 2I and S1 Table).

### *Smyd1* Tg-CKO embryos exhibit mid-gestational proliferative defects, upregulation of ER/oxidative stress genes and lethality

**Fig 3:**
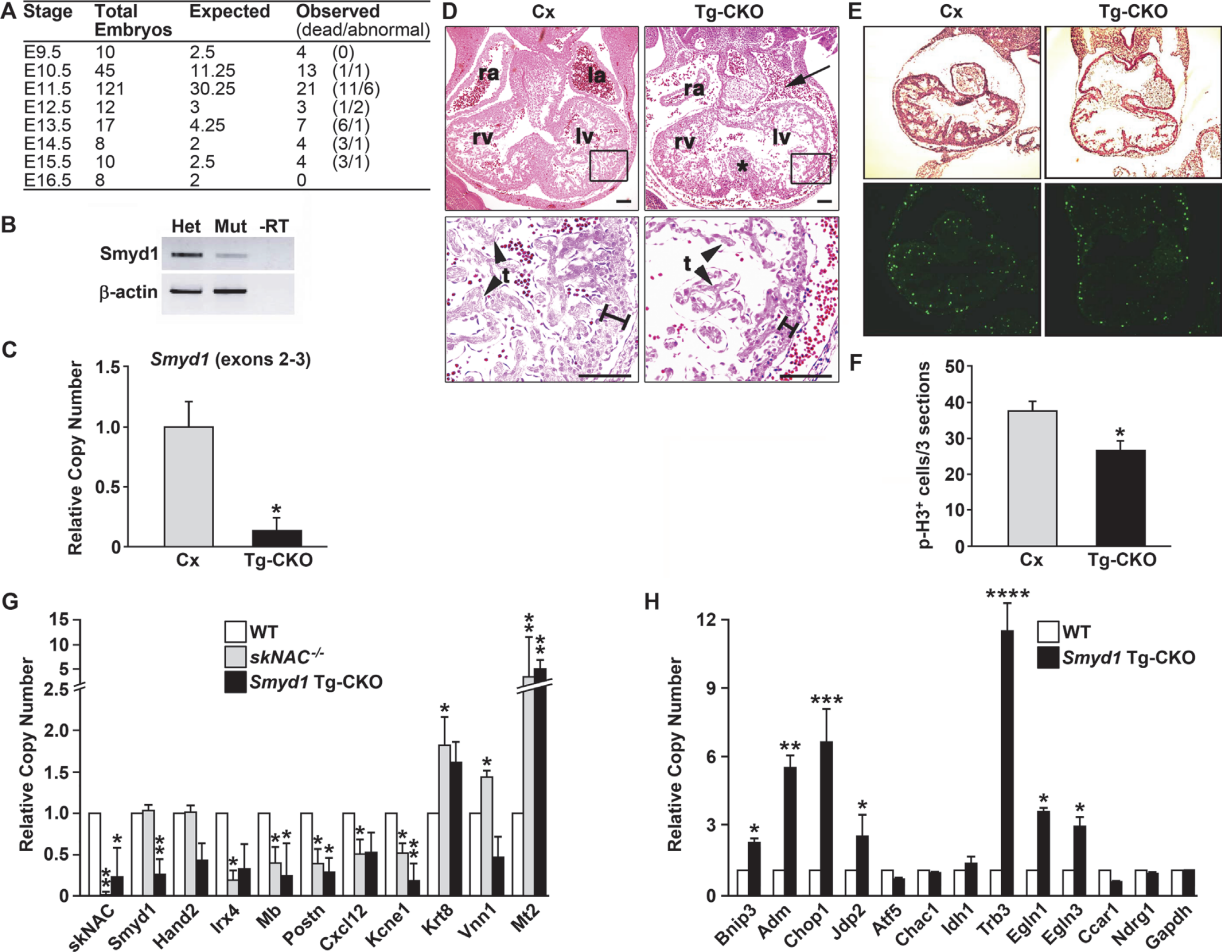
Deletion of *Smyd1* by *Tg-Nkx2*.*5-cre* leads to a delayed embryonic lethal cardiac phenotype. **A.** *Smyd1* ^*flox/flox*^; *Tg-Nkx2*.*5-cre* (Tg-CKO) embryos die at midgestation. Table numbers are total recovered embryos of each genotype. The number of dead or abnormal embryos is given in parentheses. **B, C.** *Smyd1* mRNA expression at E10.5 assayed by RT-PCR (B) and real-time PCR (C). **D.** H&E-stained transverse sections of E11.5 control (Cx) and Tg-CKO embryos showing pericardial edema, thinned pericardium and decreased trabeculation. **E.** Decreased proliferation was observed in the hearts of E10.5 *Smyd1* Tg-CKO embryos. Representative images of Cx and Tg-CKO hearts stained with H&E (upper panels) and the mitosis marker phospho-histone H3 serine 10 (p-H3) (lower panels). **F.** Quantification of p-H3 positive cells within the heart from three sections for three independent embryos (n = 3). **G.** Comparison of *skNAC* knockout and *Smyd1 Tg-CKO* heart gene expression by real-time PCR at E11.5. Data, focused primarily on oxidative response deregulation, are presented as mean for each genotype (*skNAC* ^-/-^, n = 5; *Smyd1* Tg-CKO, n = 4). Error bars indicate SEM. **H.** Genes encoding mediators of ER stress are deregulated by loss of Smyd1. Real-time PCR data represents average of 3 biological replicates each with 3 technical replicates; error bars indicate SEM. Data were analyzed by Student’s t-test (*P < 0.05, **P <0.01, ***P < 0.001, ****P < 0.0001).

In order to identify potential defects in later cardiomyocyte development, we generated a CKO of *Smyd1* using a transgenic (*Tg*)-*Nkx2*.*5-cre* line that initiates deletion primarily in differentiated ventricular cardiomyocytes [25]. Embryos were recovered at various gestational time points to record gross abnormalities and viability. By E12.5, all Tg-CKO embryos were either dead or abnormal. No live Tg-CKOs were recovered after E15.5 (Fig. 3A). RT-PCR using mRNA extracted from hearts at E10.5 showed residual *Smyd1* expression, likely the result of *Smyd1* expression in cells outside of the cre-expressing zone, such as OFT myocardium (Fig. 3B). *Smyd1* mRNA expression in the heart at E10.5 was also assayed by real-time PCR. *Smyd1* mRNA levels were significantly reduced in the Tg-CKO (Fig. 3C).

Histological analyses were conducted using embryos with detectable heartbeats upon dissection at E11.5. Serial transverse sections were used for H&E staining. Compared to control embryos, all Tg-CKO embryos (*n* = 3) exhibited pericardial edema, thinned pericardium and decreased trabeculation in similar heart sections (Fig. 3D). As observed for the Ki-CKO at E9.5, E11.5 *Smyd1* Tg-CKO embryos exhibited a significant decrease in proliferation within the heart as determined by immunofluorescence staining for the mitosis marker phospho-histone H3 S10 (Fig. 3E, F).

The observed mid-gestation *Smyd1* Tg-CKO phenotype (thinned myocardium and poor trabeculation) is similar to that resulting from the knockout of *skNAC*, a Smyd1-interacting transcription factor exclusively expressed in striated muscle and initially characterized as a positive regulator of *Mb* [9]. Smyd1 directly associates with skNAC via binding of the skNAC PXLXP motif to the Smyd1 MYND domain [11,12]. Thus, we hypothesized that the *Smyd1* Tg-CKO and *skNAC* KO exhibit similar dysregulation of gene expression. To this end, we analyzed expression of genes previously shown to be deregulated in *skNAC* knockouts at E11.5 [12] (Fig. 3G). Three of the 5 genes down-regulated in the *skNAC* knockout were also down-regulated in the *Smyd1* Tg-CKO. Of the 3 genes upregulated in the *skNAC* knockout, only *Metallothionein* (*Mt2*) was significantly upregulated. The most significantly deregulated category was oxidative stress response [4,5,8,9,12]. Strong upregulation of *Mt2* in the *Smyd1* Tg-CKO may be in response to reduced expression of *Mb* and *Vnn1*. High upregulation of *Mt2* in both animal models suggests that impaired oxidative stress defense systems may have contributed to the similar phenotype and timing of death. Expression of *skNAC* was decreased in the *Smyd1* Tg-CKO, but loss of *skNAC* did not affect expression of *Smyd1*. Thus, Smyd1 is necessary for proper expression of *skNAC*, but not vice versa. *Hand2* was down-regulated in the *Smyd1* Tg-CKO, but unaffected in the *skNAC* ^-/-^. This result supports the idea that Smyd1 and skNAC work in both complementary and independent pathways [12].

Based on previously observed similarities in genes modulated by stress response to both ER and oxidative stress [26,27,39,40,41,42], we analyzed potential deregulation in the *Smyd1* Tg-CKO of the ER stress program activated in Ki-CKO hearts (Fig. 2I). As shown in Fig. 3H, real-time RT-PCR confirmed upregulation of *Bnip3*, *Adm*, *Chop1*, *Jdp2*, *TRB3*, *Egln1* and *Egln3*. *TRB3*, a sensor for both ER and oxidative stress, exhibited the highest fold change. As with Ki-CKO hearts, we observed no apoptosis (data not shown) associated with Tg-CKO proliferative loss, and accordingly, *Ccar1* was down-regulated (p<.061) [36,37]. In contrast to the Ki-CKO ER stress response, expression of *Atf5*, *Chac1*, *Idh1* and *Ndrg1* was unaffected. These data support the idea that both the ER and the oxidative stress response is initiated in the absence of Smyd1 at both gestational stages.

### Smyd1 methylation of TRB3 provides a potential co-regulatory circuit for cardiac ER/oxidative stress transcriptional control

**Fig 4:**
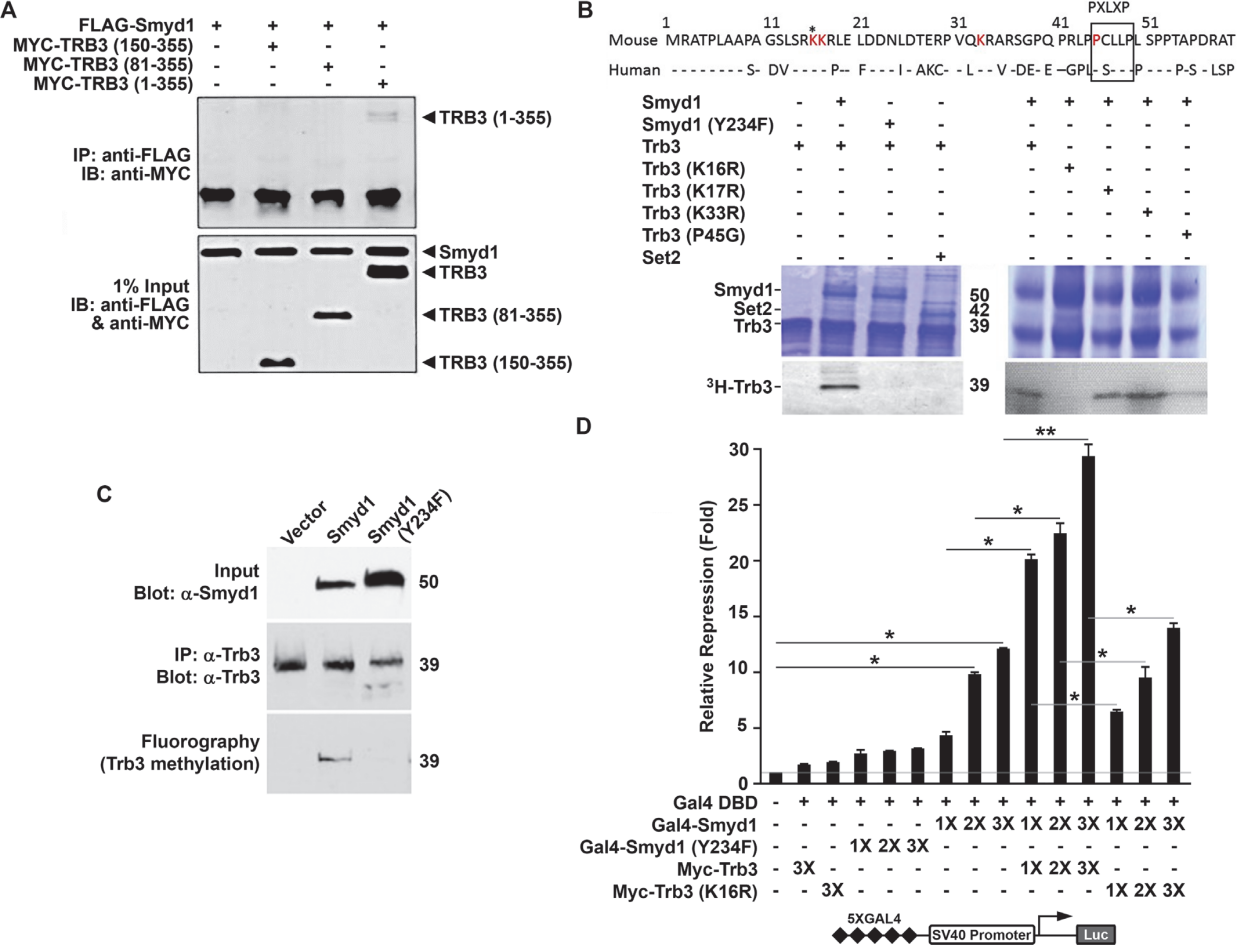
Smyd1 interacts with and methylates the ER and oxidative stress sensor Trb3 to regulate to regulate transcription. **A.** Smyd1 interacts with the Trb3 N-terminus. MYC-Trb3 and FLAG-Smyd1 were co-transfected into Cos7 cells, and cell lysates were immunoprecipitated using a monoclonal anti-FLAG M2 antibody. Following SDS-PAGE, proteins were analyzed by western blot using a polyclonal anti-MYC A14 antibody. Input is shown in the lower panel. **B.** Smyd1 *in vitro* methylation of TRB3 at K16 requires an intact MYND interaction domain. Upper panel: Comparison of N-terminal amino acid sequences of human and mouse Trb3 (dashes indicate identity) to illustrate conserved lysines (in red) and the PXLXP motif (boxed). Asterisk (*) denotes the site of methylation. Lower panels: *In vitro* methylation assays were performed using, as enzymes, recombinant 6XHis-Smyd1, 6XHis-Smyd1 catalytic mutant (Y234F) or 6XHis-Set2. Substrates were wild-type 6XHis-TRB3 or the indicated K/R substitution mutant. S-[methyl-^3^H]adenosyl-L-methionine was employed as the methyl donor. Above: Coomassie blue-staining after fractionation by 12% SDS-PAGE. Molecular weights (kD) are indicated to the side of the image. Below: Fluorography-visualized methylation of Trb3 and mutants other than Trb3-K16/R or Trb3-P45/G by Smyd1 but not Smyd1^Y234F^ or Set2. **C.** Smyd1 methylates Trb3 *in vivo*. COS7 cells were transiently transfected with MYC-Smyd1^WT^ or MYC-Smyd1^Y234F^. Two days after transfection, cells were labeled with L-[methyl-3H]-methionine in the presence of protein synthesis inhibitors. Trb3 was immunoprecipitated by anti-Trb3 antibody resolved by SDS-PAGE, pull-down confirmed by anti-Trb3 western blot (middle panel) and its methylation was detected by fluorography (lower panel). Top panel: Western blot for Smyd1 using 10% of the input used for immunoprecipitation. **D.** Methylation-competent Trb3, but not a methylation-deficient mutant (Trb3-K16/R) enhances repression by Smyd1. Cos7 cells were co-transfected with an SV40-driven luciferase reporter containing 5X-GAL4 DNA-binding domain elements, GAL4-Smyd1, RSV-lacZ and increasing amounts of the indicated MYC-Trb3 or MYC-Trb3-K16/R expression plasmid. Results were normalized to β-galactosidase activity and fold-repression (y-axis) was determined relative to SV40-driven luciferase activity alone. Data were analyzed by Student’s t-test (*P < 0.05, **P <0.01).

TRB3 is a kinase domain-containing multifunctional protein expressed broadly within the cytoplasm and nucleus of numerous cell types. While it was first characterized as a suppressor of AKT signaling [26,27], TRB3 expression is induced by several ER and oxidative stressors [26,27], and its transcription is activated by Chop1 [43], which as with TRB3, is strongly up-regulated by both *Smyd1 Ki-CKO* and *Tg-Smyd1* CKO (Fig. 2I and 3H). TRB3 was of further interest because we had previously identified it as a putative Smyd1-binding partner in yeast two-hybrid assays (data not shown). Co-immunoprecipitation experiments confirmed that Smyd1 associates with TRB3 (Fig. 4A) and that the TRB3 far N-terminal region (residues 1–80) is both necessary and sufficient for the interaction (Suppl. S2 Fig.). This previously uncharacterized N-terminal region contains a conserved MYND domain binding motif (^45^P-X/A-L-X/L-P^49^) [13,14,15]. A similar motif within skNAC is essential for its interaction with SMYD1 [11].

Smyd1 was initially characterized as a histone H3K4-specific HMTase [16]; however, non-histone substrates are also likely because Smyd1 localizes to both the nucleus and sarcoplasm [11] (Fig. 1C). Non-histone, lysine-methylated targets for its highly related paralogue, Smyd2, include the proliferation suppressors, Rb and p53 [21,22,23]. These observations prompted us to carry out *in vitro* methylation assays, employing bacterially-expressed and purified 6X-His-Smyd1 and 6XHis-TRB3 along with several TRB truncations using ^3^H-S-adenosyl-methionine as a methyl donor. Smyd1, but not an HMTase SET domain catalytic mutant (Smyd1-Y234F), methylated full-length TRB3(1–355) as well as the TRB3(1–80) N-terminus but not further C-terminal truncations (S3 Fig.). TRB3(1–80) contains three lysines (K16,17 and 33), and the former two are conserved among human and other mammals (Fig. 4B, top panel and data not shown). We mutated each of these residues as well as the P45 of the PXLXP motif within the full length TRB3(1–355) backbone and tested the purified 6X-His-proteins for methylation. Catalytically active Smyd1, but not Smyd1-Y234/F nor an unrelated HMTase SET2, robustly methylated each of the TRB3 substrates with the exception of TRB3-K16 and P45 (Fig. 4B). Thus, Smyd1 methylation of TRB3 at K16 requires PXLXP-mediated interaction of the enzyme and substrate.

To confirm that Smyd1 methylates endogenous TRB3 within mammalian cells, we utilized an assay based on the fact that the methyl group of the methyl donor S-adenosyl-methionine is derived from free methionine in the cell [32]. Two days after transfection of Smyd1-negative Cos7 cells with Smyd1 or Smyd1-Y/234/F, cells were labeled with L-[methyl-^3^H]-methionine in the presence of protein synthesis inhibitors. TRB3 was immunoprecipitated, and the immunoprecipitates were resolved by SDS-PAGE, blotted with the corresponding antibodies, and methylated proteins were detected by fluorography. As shown in Fig. 4C, wild type, but not catalytically inactivate Smyd1-Y/234/F, methylates TRB3 *in vivo*.

To test if there is a link between Smyd1 modulation of gene expression and its methylation of TRB3, we utilized a GAL4 two-hybrid assay previously established for Smyd1-mediated repression by Sims et al [11]. Transfection of GAL4-Smyd1 led to robust, dose-dependent repression of SV40-driven 5x-GAL4-DBD reporter activity when analyzed 48 hr following transfection into Cos7 cells. Co-transfection of Myc-tagged-TRB3 full-length, but not the Myc-TRB3-K16/R methylation-deficient substitution mutant, led to significantly enhanced repression in a dose dependent manner (Fig. 4D). Thus, methylated TRB3, at least in cultured cells, functions as a specific co-repressor for Smyd1, suggesting a possible feedback loop to control *TRB3* transcription and putatively other genes underlying the stress response to Smyd1 loss.

## Discussion

To gain insight into the physiological functions of *Smyd1* in the developing heart, we conditionally inactivated the *Smyd1* gene using two different *Nkx2*.*5-cre* drivers, which become active in early cardiomyocyte progenitors (*Ki-Nkx2*.*5-cre*) or primarily in differentiated ventricular cardiomyocytes (*Tg-Nkx2*.*5-cre*). Our findings indicate that *Smyd1* is a critical regulator of cardiac development at both the stage at which the SHF gene program is initiated and at later stages of cardiac morphogenesis. Both CKOs exhibit impaired cell proliferation and activation of stress pathways, which may be consequence of proliferative failure. A component of the underlying molecular mechanism of Smyd1 is upregulation and subsequent methylation of TRB3, a sensor for both ER and oxidative stress and suppressor of Akt signaling [26,27]. Smyd1 and TRB3 act synergistically to repress transcription in a methylation-dependent manner.

### Different phenotypes with different *Nkx2*.*5-cre* drivers

It is of particular interest that the *Smyd1* CKO phenotypes using two different *Nkx2*.*5-cre* drivers were dissimilar. Ki-CKO embryos died by E10.5 with impaired expansion of the SHF. Tg-CKO embryos survived until E11.5 or later, showed normal development of SHF-derived structures and exhibited myocardial abnormalities in both ventricles. The knock-in *Nkx2*.*5-cre* disrupts the *Nkx2*.*5* open reading frame [24]. As a consequence, the *Smyd1* Ki-CKO is also heterozygous for *Nkx2*.*5*. It is possible that loss of one *Nkx2*.*5* allele amplifies the loss of *Smyd1* phenotype; however, the difference in phenotype between the two CKOs is more likely due to differences in spatial and temporal expression of the two *Nkx2*.*5-cre* drivers. The transgenic *Nkx2*.*5-cre* [25] is fused to an enhancer that directs expression primarily in ventricular cardiomyocytes, whereas the knock-in *Nkx2*.*5-cre* employs the full range of endogenous enhancer/promoter elements and is active as early as E7.5 in the cardiac crescent [24]. Since the enhancer driving *Tg-Nkx2*.*5-cre* has a more restricted expression pattern than the endogenous *Nkx2*.*5* gene [44], it is likely that Tg-CKO embryos survive longer due to delayed deletion of *Smyd1* or lack of deletion in SHF progenitors and OFT myocardium.

*Smyd1* Ki-CKO hearts exhibited similarities with those of the conventional *Smyd1* knockout [10], whereas the *Smyd1* Tg-CKO phenotype was more similar to that of the *skNAC* knockout [12]. The conventional *Smyd1* knockout failed to form a RV, showed reduced expression of SHF genes and died before E10.5. Likewise, the *Smyd1* Ki-CKO showed reduced size of SHF-derived structures (OFT and RV), a reduction is SHF gene expression and lethality by E10.5. The striated muscle-restricted transcription factor skNAC is one of the few known Smyd1-interacting proteins [9,11]. The *skNAC* knockout and *Smyd1* Tg-CKO both exhibited myocardial defects in both ventricles, including thinning of the compact myocardium and decreased trabeculation. Furthermore, many of the same genes dysregulated in the *skNAC* knockout were similarly dysregulated in the *Smyd1* Tg-CKO. These observations support the idea that the Smyd1-skNAC interaction regulates cardiomyocyte gene expression and is important for proper heart development.

### Alterations in SHF-related gene expression

Among the 803 annotated transcripts significantly deregulated by *Smyd1* elimination at E9.5, 324 were down-regulated, while 499 were upregulated. *Smyd1* loss altered gene expression of a number of pathways (S1 Table), but particularly relevant to the early phenotype were genes regulating SHF development/maintenance. Prominent among these is the SHF transcription factor *Isl1*, whose expression defines the SHF and is critical for its proper differentiation [1,2]. Mice lacking *Isl1* expression die *in utero* due to severe heart malformation, lacking all derivatives of the SHF [45,46,47]. Thus, reduction of *Isl1* gene expression in *Smyd1* Ki-CKO hearts could explain why the OFT and RV were reduced in size. Isl1 drives expression of core SHF cardiac transcription factors, including *Mef2c* [48]. The *Smyd1* gene is a direct target of Mef2c [49]. Reduction in *Isl1* expression suggests a positive feedback loop from *Isl1* to *Mef2c* to *Smyd1* and back to *Isl1*. In support, expression of *Mef2c* was also reduced in *Smyd1* Ki-CKO hearts. Since residual *Isl1* remained restricted to the pharyngeal mesoderm, additional factors not regulated by Smyd1 must control its spatial expression pattern.

Although expression of *Hand2*, *Mef2c*, *Shox2*, *Tbx1* and *Isl1* were reduced in *Smyd1* Ki-CKO embryos, none were completely silenced. Thus, the severe SHF phenotype resulting from loss of *Smyd1* may result from the combined effects of reducing expression of each of these genes as well as other genes comprising pathways not restricted to SHF. For example, we observed deregulation of chamber-specific factors (e.g. *Myh7*, *Bmp7*, *Anf*, *Acta1*, *Egr1 and Fos)* that are also upregulated in adult cardiac hypertrophy and/or heart failure [50,51,52,53]. While hypertrophy is unlikely to be detectable in early embryonic cardiomyocytes, induced deletion of *Smyd1* in adult hearts leads to marked hypertrophy with upregulation of the same markers (Franklin et al, *in review*).

### Alterations in expression of ER and oxidative stress genes

In the absence of Smyd1, cardiomyocyte proliferation is reduced concurrent with upregulation of genes involved in ER and oxidative stress responses. Gene expression profiling at E9.5 uncovered an apparent attempt to resolve ER stress via autophagy. Cells exposed to ER stress undergo the unfolded protein response in an attempt to avoid apoptosis, but may also activate autophagy [6,7]. While dysregulation of autophagy has been implicated in multiple pathological conditions, including cardiovascular diseases [54,55], little is known as to regulation of this pathway in the developing heart. In the *Smyd1* Ki-CKO, three key trans-activators of the autophagic pathway were upregulated, including *BCL2/adenovirus E1B 19-kDa interacting protein 3 (Bnip3)*, *Activation Transcription Factor 5 (Atf5)*, and *Chop1/GADD153* [6,7,56]. Atf5 directly activates another gene up-regulated in the *Smyd1 Ki-CKO*, the hallmark autophagy marker, *cation transport regulator homolog 1* (*Chac1*) [6,7,56]. *Chac1* and *Idh1* are known to be differentially expressed in response to autophagic vs. apoptotic signals [36,37]. *Isocitrate Dehydrogenase 1 (Idh1)* and *Cell Cycle and Apoptosis Regulatory Protein 1 (Ccar1)* were down-regulated in the *Smyd1 Ki-CKO*. These genes are canonically down-regulated during autophagy induced by ER-stress, but are upregulated in ER stress-induced apoptosis [36,37]. This observation is consistent with the non-apoptotic phenotype observed in *Smyd1* Ki-CKO. *Prolyl hydroxylase domain 1* and -*3 (PHD/Egln1/3)* are known to be upregulated in cardiomyocytes by both ER stress and hypoxia [38], as are several additional genes which are overexpressed in the *Smyd1* Ki-CKO including, *Jun dimerization protein 2* (*Jundm2/JDP2*), *adrenomedullin* (*Adm*), and *Tribbles 3* (*TRB3*) [26,27,39,40,41].

Although the above gene expression deregulation suggests initiation of autophagic resolution to ER stress, *Smyd1* Ki-CKO cardiomyocytes did not show morphological signs of cellular degradation characteristic of the execution phase of this mechanism. This second stage is mediated by the autophagic PERK/eIF2α axis which, at least by transcript profiling, was unaffected in *Smyd1* Ki-CKO (data not shown) [57]. This apparent paradox may be rationalized by the parallel strong upregulation of *N-myc down-regulated gene 1* (*Ndrg1*) [58] in the *Smyd1* Ki-CKO. *Ndrg1* over-expression inhibits the ER stress-mediated autophagic pathway by suppressing PERK/eIF2α leading to a pro-survival phenotype [59]. As a further link between the observed early and late phenotypes of ER and oxidative stress, *Idh1* reduction as well as *Egln1* and *Egln3* upregulation leads to decreased intracellular NADPH and, thus, to an increase in intracellular ROS [38]. Such triggers may underlie the array of oxidative stress response mediators shared by Smyd1 and skNAC-deficiencies.

In response to hypoxia and numerous stresses, tissues implement mechanisms to enhance oxygen delivery, including the activation of angiogenesis [52]. *Ndrg1* and *Adm*, upregulated targets discussed above in the context of ER stress, are also strong inducers of angiogenesis. Several additional genes upregulated by *Smyd1* Ki-CKO belong to the angiogenesis pathway. These include *VegFα*, *Plek*, and *CxCl4* (S1 Table).

### A potential Smyd1-TRB3 feedback loop for stress response regulation

We identified TRB3 as a non-histone methylation target of Smyd1. TRB3 and its family members function at the intersection of multiple stress-activated pathways including the mammalian target of rapamycin (mTOR), ER stress, oxidative stress and autophagy [60]. Consistent with a functional role in *Smyd1* deficiency-mediated anti-proliferative responses, *TRB3* transcript levels were significantly up-regulated in both Ki-CKO and Tg-CKO hearts. We confirmed TRB3 to be a Smyd1-interacting protein and found that Smyd1 methylates TRB3 *in vitro* and *in vivo*. Methylation of TRB3 at a single conserved lysine in its previously uncharacterized N-terminal 80 amino acids requires an intact PXLXP motif, which is known to mediate interactions with MYND domains. Thus, Smyd1 interacts with TRB3 through its MYND domain and methylates TRB3 through its SET domain. Methylated, but not unmethylated, TRB3 functions as a co-repressor for Smyd1.

Collectively these data indicate that Smyd1 regulates oxidative and ER stress responses at two levels. First, Smyd1 activates *TRB3* transcription, perhaps indirectly via activation of *Chop1*, which encodes an established trans-activator of *TRB3* [43]. Second, Smyd1 converts TRB3 into a co-repressor via direct methylation. The feasibility of this feedback loop is strengthened by the previously observed nucleo-cytoplasmic shuttling of Smyd1 during myocyte differentiation [11]. It remains an important extension of this work to determine to what extent target gene dysregulation in the absence of Smyd1 is due to direct epigenetic targeting of gene regulatory regions or due to methylation of non-histone proteins.

### Concluding remarks

In summary, we have shown that *Smyd1* is essential for proper expression of the cardiac gene program. *Smyd1* null mutants exhibit malformation of SHF derivatives owing, at least in part, to reduced expansion of the progenitor pool. Smyd1 also regulates response to ER and oxidative stress. In the absence of Smyd1, a gene expression program consistent with a non-apoptotic autophagic response is initiated. These gene expression changes are consistent with observed reduction in cell proliferation and the absence of apoptosis. We also have shown that later in development, Smyd1 continues to play an essential role in response to oxidative stress, through both interaction with skNAC and dysregulation of non-skNAC target genes. As *Smyd1* is continuously expressed into adulthood, it is likely involved with these pathways in both normal and pathologic conditions. For example, during ischemia as well as development and progression of maladaptive myocardial remodeling and failure, there is significant nutrient and oxygen starvation which leads to the induction of a number of ER and oxidative stress response genes—several of whom we observed here to be deregulated by Smyd1 deficiency [61,62,63]. Further analysis of Smyd1 function in adult tissues could lead to a better understanding of these mechanisms and allow development of novel and effective therapeutic strategies against heart failure.

## Supporting Information

S1 Fig Construction of the *Smyd1* ^*flox*^ conditional allele and initial analyses following *Nkx2*.*5-cre* deletion in the developing heart. **A.** Illustration of the gene targeting strategy to generate the *Smyd1* conditional knockout allele. The *neo* cassette was subsequently removed via breeding to *EIIA-cre* and selecting for loss of *neo* and retention of exons 2–3 (*Smyd1* ^*flox*^ allele). **B.** Confirmation of correct *Smyd1* gene targeting by Southern blot. Southern blots were performed using probes flanking the left and right homology arms (probes 2 and 3) to distinguish the 13.7 kb and 4.5 kb wild type (WT) allele fragments from the targeted allele (7.5 and a 7.0 kb), respectively. **C.** *Smyd1 flox* and *WT* alleles were distinguished by PCR. **D, E.** No *Smyd1* RNA (D) or protein (E) was detectable in *Smyd1* ^*flox/flox*^;*Ki-Nkx2*.*5-cre* (Ki-CKO) hearts at E9.5.
(TIF)

S2 Fig The N-terminus of Trb3 is necessary and sufficient to interact with Smyd1. C-terminal MYC-Trb3 deletion constructs were tested by co-immunoprecipitation with FLAG-Smyd1 to map the region of Trb3 that interacts with Smyd1. Cos7 cells were co-transfected FLAG-Smyd1 and the MYC-Trb3 deletion constructs, incubated for 30 h and lysed. Cell lysates were immunoprecipitated using a monoclonal anti-FLAG M2 antibody. The immunoprecipitates were separated by SDS-PAGE and analyzed by western blot using a polyclonal anti-MYC A14 antibody. Input is shown in the lower panels. The far N-terminal deletion (1–80) was sufficient for interaction with Smyd1.
(PDF)

S3 Fig Smyd1 methylates a site within the N-terminal 80 amino acids of Trb3. 6X-His-tagged wild-type Trb3(1–355) and the indicated truncation mutants were tested as substrates for methylation by full-length Smyd1. Methods are detailed in Materials and Methods section and legend to Fig. 4B.
(TIF)

S1 Table Genes mysregulated in *Smyd1* Ki-CKO hearts. Annotated genes were organized based on gene ontology using DAVID software [64]. These data were derived from two independent biologic replicas and at least 2 technical replicas of each for wild type (wt: *Nkx2*.*5* ^*+/+*^; *Smyd1* ^*Flox/Flox*^) and mutant (mut: *Smyd1* Ki-CKO : *Nkx2*.*5* ^*cre/+*^; *Smyd1* ^*Flox/Flox*^) hearts at E9.5. Down-regulated transcripts indicated in yellow.
(PDF)
