# Supplementary figures and images for: Smyd1 Facilitates Heart Development by Antagonizing Oxidative and ER Stress Responses
Source: PLoS One. 2015 Mar 24;10(3):e0121765. doi: 10.1371/journal.pone.0121765 (PMC4372598; doi:10.1371/journal.pone.0121765)

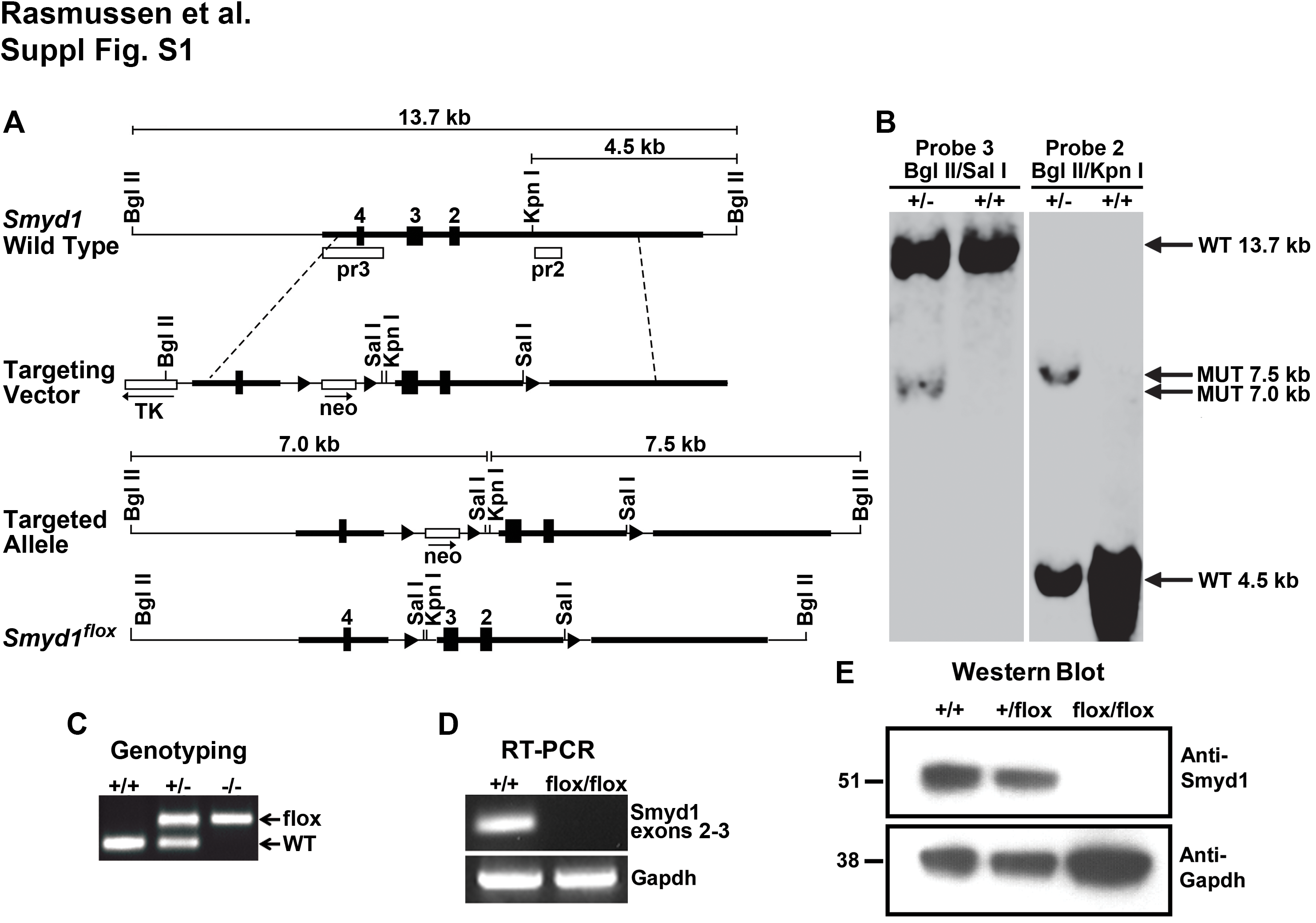

Supplement: S1 Fig — A. Illustration of the gene targeting strategy to generate the Smyd1 conditional knockout allele. The neo cassette was subsequently removed via breeding to EIIA-cre and selecting for loss of neo and retention of exons 2–3 (Smyd1 flox allele). B. Confirmation of correct Smyd1 gene targeting by Southern blot. Southern blots were performed using probes flanking the left and right homology arms (probes 2 and 3) to distinguish the 13.7 kb and 4.5 kb wild type (WT) allele fragments from the targeted allele (7.5 and a 7.0 kb), respectively. C. Smyd1 flox and WT alleles were distinguished by PCR. D, E. No Smyd1 RNA (D) or protein (E) was detectable in Smyd1 flox/flox;Ki-Nkx2.5-cre (Ki-CKO) hearts at E9.5. (TIF) [file pone.0121765.s001.tif]

Rasmussen et al.  
Suppl. Fig. S2

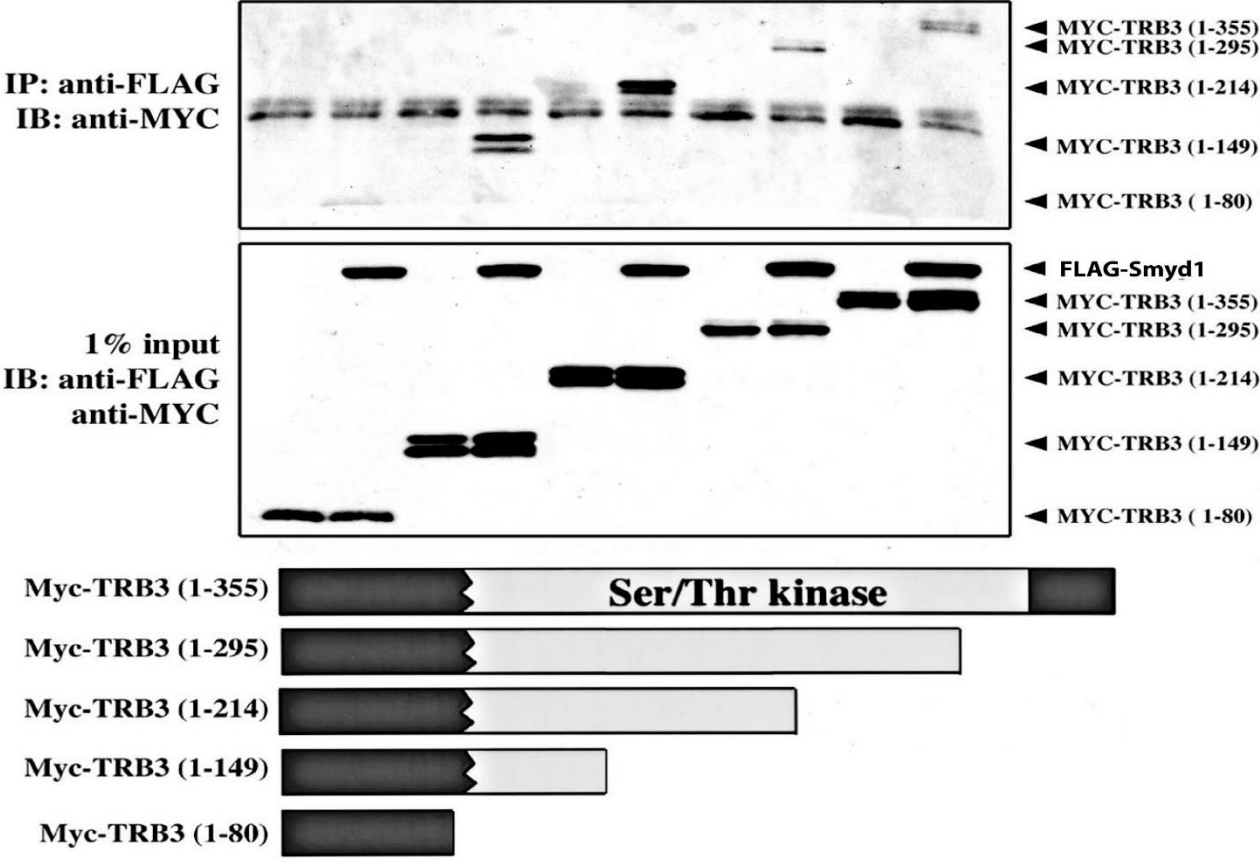

Supplement: S2 Fig — C-terminal MYC-Trb3 deletion constructs were tested by co-immunoprecipitation with FLAG-Smyd1 to map the region of Trb3 that interacts with Smyd1. Cos7 cells were co-transfected FLAG-Smyd1 and the MYC-Trb3 deletion constructs, incubated for 30 h and lysed. Cell lysates were immunoprecipitated using a monoclonal anti-FLAG M2 antibody. The immunoprecipitates were separated by SDS-PAGE and analyzed by western blot using a polyclonal anti-MYC A14 antibody. Input is shown in the lower panels. The far N-terminal deletion (1–80) was sufficient for interaction with Smyd1. (PDF) [file pone.0121765.s002.pdf]

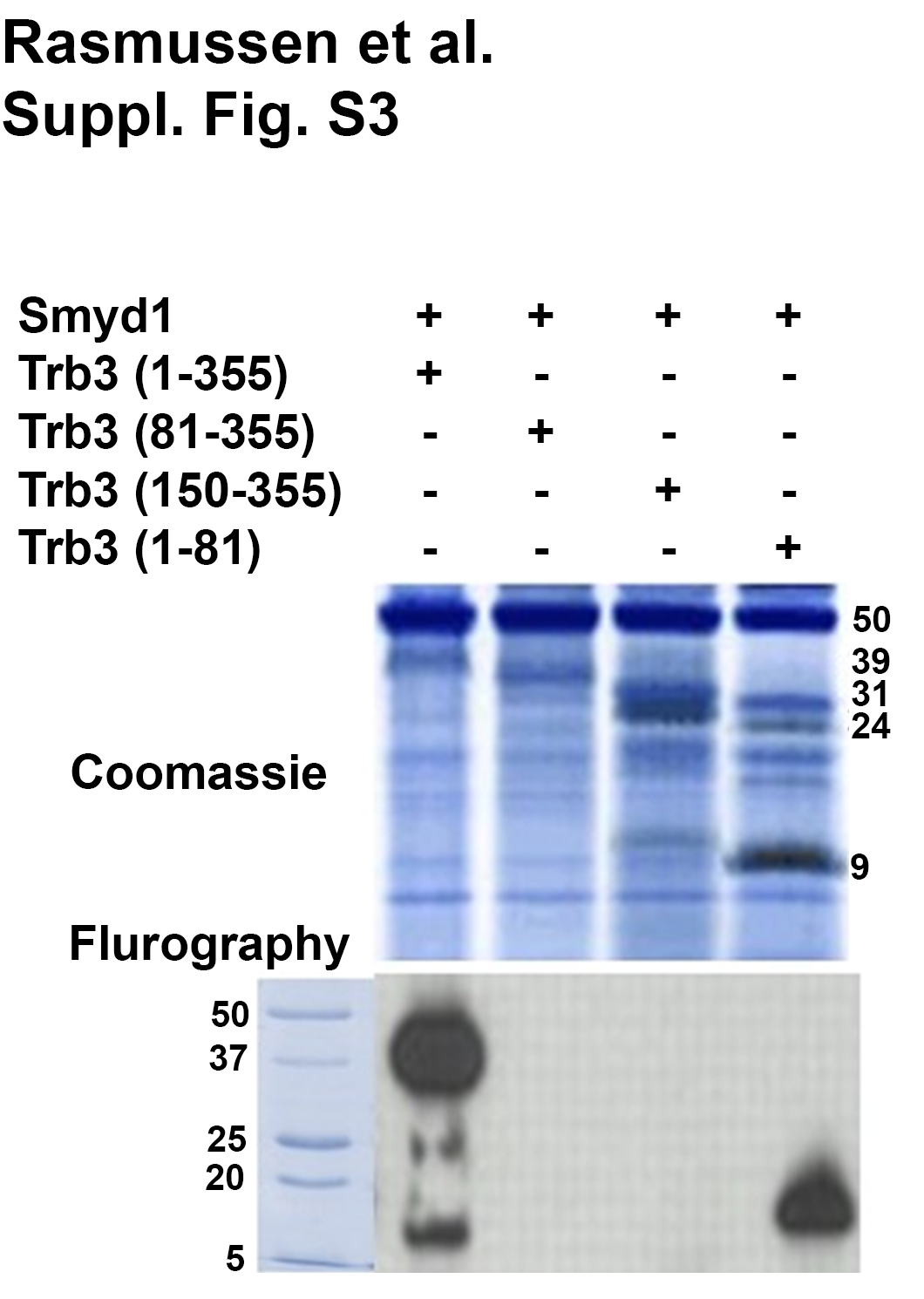

Supplement: S3 Fig — 6X-His-tagged wild-type Trb3(1–355) and the indicated truncation mutants were tested as substrates for methylation by full-length Smyd1. Methods are detailed in Materials and Methods section and legend to Fig. 4B. (TIF) [file pone.0121765.s003.tif]
