# Supplementary material for: Smyd1 Facilitates Heart Development by Antagonizing Oxidative and ER Stress Responses
Source: PLoS One. 2015 Mar 24;10(3):e0121765. doi: 10.1371/journal.pone.0121765 (PMC4372598; doi:10.1371/journal.pone.0121765)
Supplement: S1 Table — Annotated genes were organized based on gene ontology using DAVID software [64]. These data were derived from two independent biologic replicas and at least 2 technical replicas of each for wild type (wt: Nkx2.5 +/+; Smyd1 Flox/Flox) and mutant (mut: Smyd1 Ki-CKO : Nkx2.5 cre/+; Smyd1 Flox/Flox) hearts at E9.5. Down-regulated transcripts indicated in yellow. (PDF) [file pone.0121765.s004.pdf]

Rasmussen et al.

Supplementary Table S1

ECM and Structure	mut1 vs wt1	mut2 vs wt2
Down regulated genes		
Reln	0.64	0.25
Tppp3	0.36	0.38
Mfap4	0.5	0.38

Skeletal Muscle	mut1 vs wt1	mut2 vs wt2
Up regulated genes		
Tcf15	2.48	2.46
Mib2	1.89	2.12
Down regulated genes		
Phox2b	0.313	0.605
Barx1	0.515	0.247

Proliferation, ER Stress, and Autophagy	mut1 vs wt1	mut2 vs wt2
Up regulated genes		
Chac1	15.6	16.2
Trib3	5.42	4.82
Ndrg1	4.94	4.16
Lims2	5.33	4.09
Atf5	2.35	3.12
Jundm2	3.05	2.85
Igfbp2	2.88	2.39
Bnip3	1.85	2.83
Otub2	2.47	3.02
Eif4ebp1	2.47	2.44
Stag3	2.06	1.66
Nfil3	2.07	1.47
Jmjd6	1.62	1.66
Atraid	1.54	2
Dab2	1.52	2.07
Ccnl2	1.47	3.11
Down regulated genes		
Pik3r3	0.33	0.37
Afp	0.2	0.04
Idh1	0.47	0.23
Ubtf	0.58	0.42
Ccar1	0.69	0.4
Mtvr2	0.57	0.43

Angiogenesis and Hemotapoiesis	mut1 vs wt1	mut2 vs wt2
Up regulated genes		
Adm	4.96	7.19
Alas	1.6	1.6
Egln1	3.86	3.8
Egln3	6.75	5.9
Plek	3.53	3.39
Vegfa	4.01	6.28
Egr1	1.91	2.42
Cxcl4	8.63	3.64
Efna1	1.45	1.87
Down regulated genes		
Vtn	0.25	0.21

Cardiogenesis	mut1 vs wt1	mut2 vs wt2
Up regulated genes		
Acta1	3.08	6.08
Muc2	3.61	6.61
Hspa1b	4.14	3.13
Pdk1	3.74	2.32
Dkk3	2.37	2.73
Zic3	2.36	2.27
Nkx2.3	1.45	1.42
Obscn	2.42	2.03
Ftcd	1.88	2.26
Leprel1	2.26	1.93
Myh10	1.55	2.91
Tpm2	1.54	1.65
Lrrc10	2.19	1.67
Fos	2.01	2.73
Krt8	2.43	1.58
Wnt6	2.02	1.55
Mbnl2	1.51	2.54
Pax8	1.43	2.32
Snail3	2.54	1.8
Arsa	2.03	1.82
Bag2	1.48	1.84
Tcf25	1.45	1.86
Atp2a2	1.6	2.07
Pgk1	1.85	2.57
Down regulated genes		
Msc	0.24	0.45
Ttr	0.52	0.16
Nkx6.1	0.44	0.56
Shox2	0.43	0.49
Mylk	0.71	0.65
Gata5	0.71	0.67
Tbx5	0.65	0.97
Foxfa1	0.6	0.32
